# First person – Karin Tuschl

**DOI:** 10.1242/dmm.049641

**Published:** 2022-06-15

## Abstract

First Person is a series of interviews with the first authors of a selection of papers published in Disease Models & Mechanisms, helping early-career researchers promote themselves alongside their papers. Karin Tuschl is first author on ‘ Loss of *slc39a14* causes simultaneous manganese hypersensitivity and deficiency in zebrafish’, published in DMM. Karin conducted the research described in this article while an academic clinical lecturer in Prof. Stephen Wilson’s and Prof. Corinne Houart's labs at University College London and King's College London, London, UK, respectively. She is now a MRC Clinician Scientist Fellow and has established her own group at UCL GOS Institute of Child Health, London, investigating the role of manganese in brain physiology and disease – from inherited manganese transporter defects to common neurodegenerative disorders.


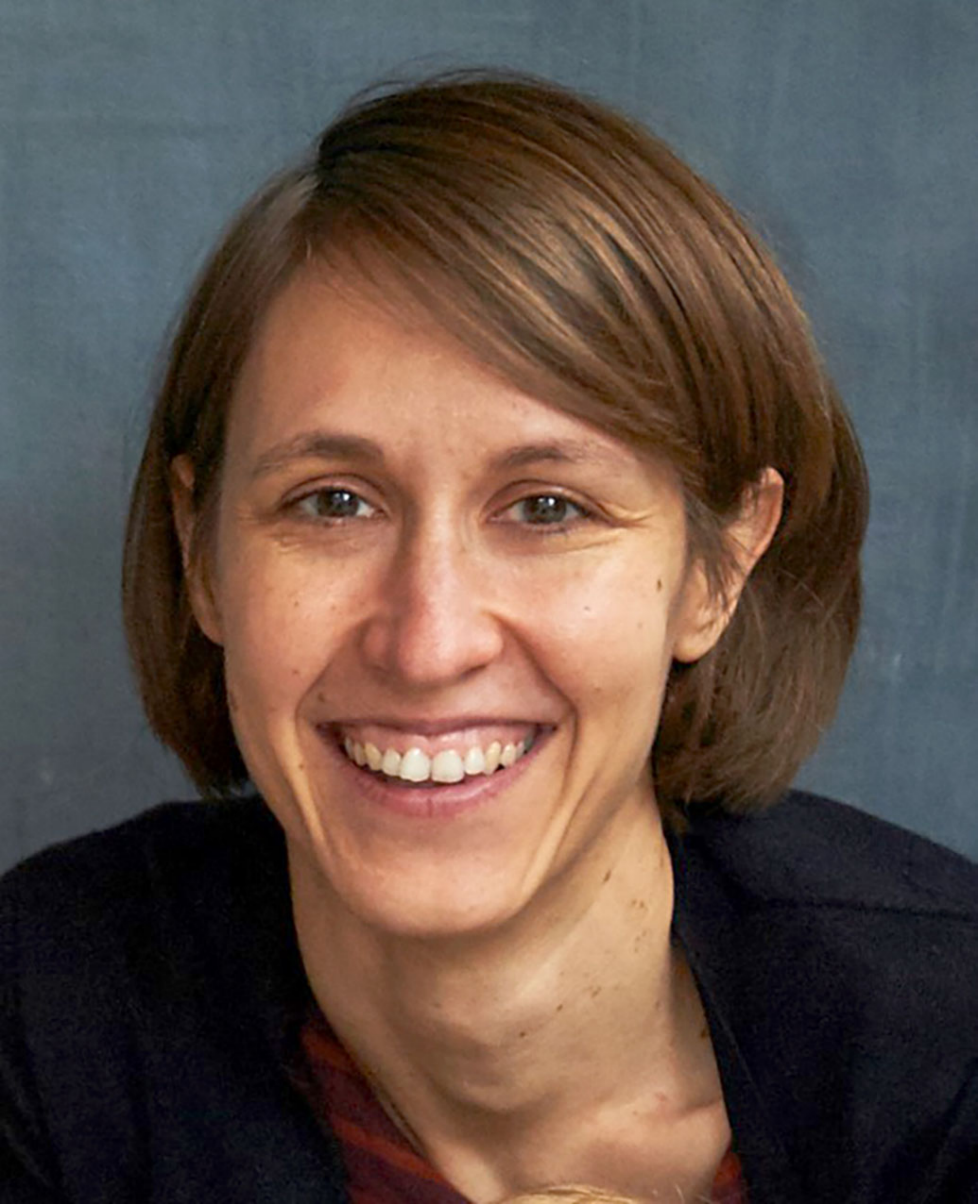


**Karin Tuschl**

**How would you explain the main findings of your paper to non-scientific family and friends?**

Manganese is an essential trace metal present in our diet that is required for normal brain function. However, exposure to high manganese concentrations, for instance, in occupational settings such as mining industries or in end stage liver disease, causes brain damage and a debilitating movement disorder similar to Parkinson's disease. We have recently identified an inherited disorder caused by abnormalities in the gene *SLC39A14* that is required for manganese transport across the cell. Impaired control of the body's manganese load results in manganese overload in the brain, which is associated with a disabling neurodevelopmental disorder in childhood. In order to study the toxic effects of manganese on the brain, we generated a zebrafish disease model by altering the *slc39a14* zebrafish gene. Mutant zebrafish recapitulate the human manganese overload disorder, resulting in impaired swimming behaviour and eye function. In this work, we assessed the changes in gene expression upon manganese toxicity. This suggested that manganese overload leads to a cellular stress response coupled with alterations in calcium levels accompanied by abnormal neuronal activity, reduced motor activity and visual impairment. In addition, we found that, in parallel to manganese overload, manganese deficiency was present, likely in certain compartments of the cell, which is consistent with disturbed manganese transport across the cell.

“Impaired control of the body's manganese load results in manganese overload in the brain, which is associated with a disabling neurodevelopmental disorder in childhood.”

**What are the potential implications of these results for your field of research?**

Our understanding of how manganese imbalance leads to disease is poor and treatments to alleviate neurological symptoms for the above conditions remain unsatisfactory. The zebrafish model we generated presents a valuable model to study the mechanisms underlying manganese toxicity in the brain and screen new treatment approaches. Our work has suggested several downstream effects of manganese toxicity with calcium disturbance and cellular stress being key events. These results provide a starting point from which to investigate disease mechanisms further with the view to identifying new therapeutic targets and improving treatments for manganese-associated disorders.

**What are the main advantages and drawbacks of the model system you have used as it relates to the disease you are investigating?**

Zebrafish have unique characteristics that make them an ideal model organism for neuroscience. They are of small size and are transparent, which allows imaging of their whole brain at single-cell resolution. Zebrafish can be analysed in high numbers and thereby are particularly suited for drug screening. As a vertebrate, zebrafish share genetic similarity with humans as their genome has a high degree of sequence and functional homology. Therefore, the majority of proteins have the same function across both species; for instance, zebrafish share the same neurotransmitter systems with humans. Zebrafish are easily amenable to genetic modifications and, hence, they are ideally suited to study the consequences of genetic mutations. This has allowed us to generate a zebrafish model of manganese neurotoxicity that recapitulates the disease features observed in humans. In addition to changes in their motor behaviour, zebrafish develop visual impairment upon manganese toxicity. We take advantage of this fact and study the effects of manganese in the retina that has similar neuronal networks as the brain, in order to better understand what happens in the brain. While zebrafish are invaluable for drug screening and candidate identification, it is unlikely that they can replace rodents in the later phases of drug discovery, which is the major drawback of zebrafish for translational studies.

**Figure:**
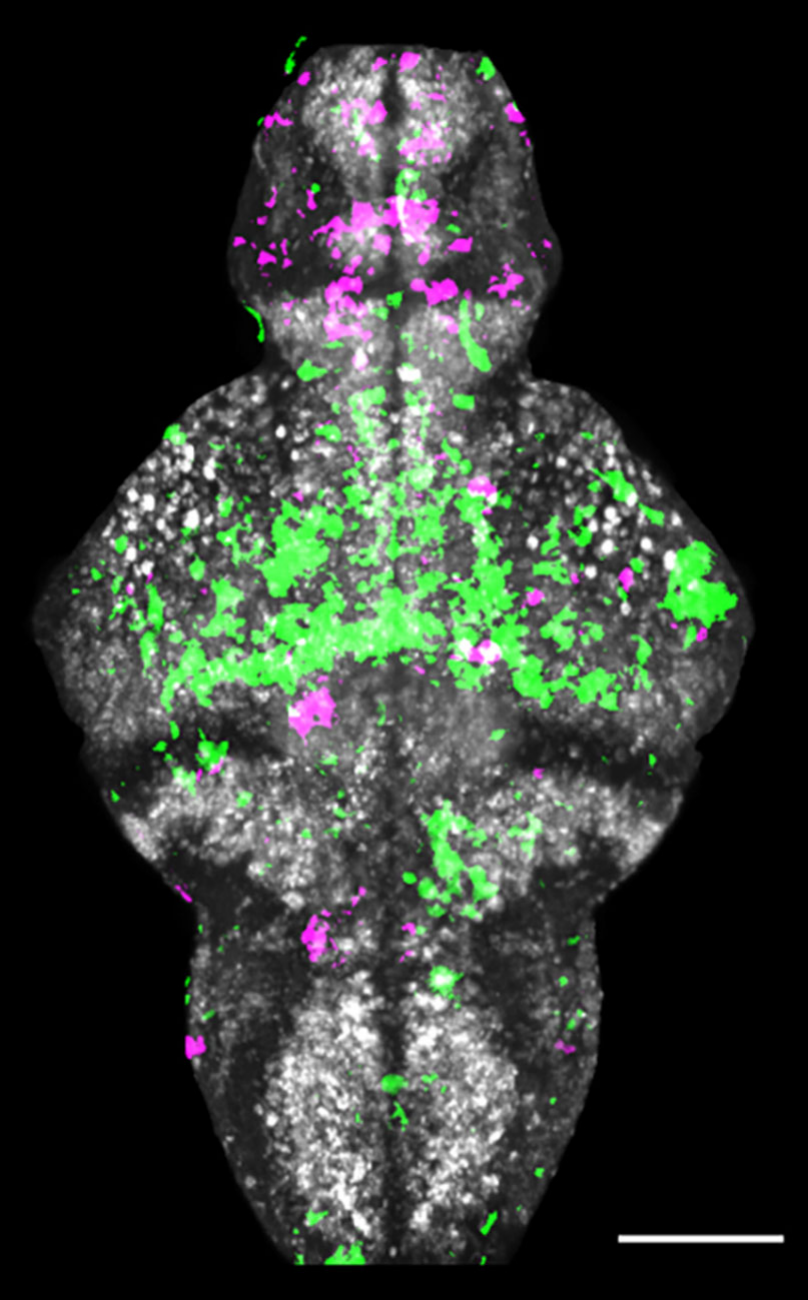
**Zebrafish brain showing changes in neuronal activity upon manganese toxicity.** Increased activity is shown in magenta, suppressed activity in green. Scale bar: 100 μm

**What has surprised you the most while conducting your research?**

Most surprising was the fact that impaired manganese transporter function in our zebrafish model seems to affect different compartments of the cell or tissues differently. In parallel to manganese accumulation and toxicity, there appears to be a degree of manganese deficiency. This teaches us a new aspect of the human disease that may have implications for treatment.

**Describe what you think is the most significant challenge impacting your research at this time and how will this be addressed over the next 10 years?**

Manganese neurotoxicity has been studied for decades and a number of downstream effects identified. However, the major challenge remains to determine the key molecular targets and elucidate hierarchical interactions between them. This is a prerequisite for the identification of new therapeutic targets. The recently identified inherited manganese transporter defects and development of associated disease models in zebrafish and mouse have opened up new avenues to investigate disease mechanisms. Hence, I am convinced that our understanding of the effects of manganese neurotoxicity will rapidly evolve and lead to novel insights into disease pathogenesis and better treatments.

**What changes do you think could improve the professional lives of early-career scientists?**

Early career researchers are faced by a wealth of challenges that include funding, publishing, competitiveness and job insecurity. Speaking as a clinical academic, these issues are complicated by the challenge of integrating research into clinical training and, ultimately, doing two jobs in parallel. While the development of integrated academic training in the UK has improved some of these issues, inflexibility around training remains. Addressing inflexibility and making training more bespoke in order to facilitate career progression for clinical academics could make a big difference and attract more clinical trainees to academia.

“I will establish […] my research to develop better treatments for manganese overload, something that is close to my heart as a clinician caring for children with rare disorders […]”

**What's next for you?**

As a newly established PI, I hope to expand my group within the next few years in order to maximise research output and attract further funding. I will establish a new and exciting translational aspect of my research to develop better treatments for manganese overload, something that is close to my heart as a clinician caring for children with rare disorders for which effective therapies are minimal. Ultimately, I wish for my research to mirror my clinical practice and establish myself as an expert in the field.
